# Anatomical Changes in a Case with Asymmetrical Bilateral Maxillary Sinus Hypoplasia

**DOI:** 10.3390/medicina58050564

**Published:** 2022-04-20

**Authors:** Adrian Cosmin Ilie, Adelina Maria Jianu, Mugurel Constantin Rusu, Alexandru Nicolae Mureșan

**Affiliations:** 1 Department III Functional Sciences, Division of Public Health and Management, Faculty of Medicine, “Victor Babeș” University of Medicine and Pharmacy, RO-300041 Timișoara, Romania; ilie.adrian@umft.ro; 2 Department I Anatomy-Embryology, Division of Anatomy and Embryology, Faculty of Medicine, “Victor Babeș” University of Medicine and Pharmacy, RO-300041 Timișoara, Romania; 3 Department I, Division of Anatomy, Faculty of Dental Medicine, “Carol Davila” University of Medicine and Pharmacy, RO-020021 Bucharest, Romania; alexandru-nicolae.muresan@drd.umfcd.ro; 4 Research Department, “Dr. Carol Davila” Central Military Emergency Hospital, RO-010825 Bucharest, Romania

**Keywords:** maxilla, cone beam computed tomography, nasal cavity, uncinate process, orbit, pterygopalatine fossa

## Abstract

*Background and Objectives*: The maxillary sinus hypoplasia (MSH) is an occasional variation of the maxilla, occurring either unilaterally or bilaterally. Previous studies dealing with MSH have not detailed the consequent anatomical changes of the maxilla and adjacent fossae. *Materials and Methods*: A 58-year-old female case was scanned in Cone Beam Computed Tomography and found to have asymmetrical bilateral MSH, who was then further evaluated anatomically. *Results*: The maxillary sinuses were hypoplastic and had mild mucosal thickenings. The orbital floors were curved. The uncinate process and the ethmoidal infundibulum were laterally displaced beneath the orbit floor. On each side, the lateral nasal wall protruded within the respective maxillary bone to reach above the vestibular cortical plate of the alveolar process. This expansion of the lateral nasal walls was limited to the premolar and first molar regions. The inferior turbinates were laterally curved. The perpendicular palatine plate was building a postero-lateral nasal wall in front of the pterygopalatine fossa. *Conclusions*: The classification systems of MSH should be detailed to indicate whether the normal uncinate process is medial or inferior to the orbit. The lateral expansion of the lateral nasal wall in MSH is limited to the anterior part of that wall. The laterally expanded nasal fossa could reach anterior to the pterygopalatine fossa in MSH. Seemingly, CBCT is a better tool than CT to evaluate the detailed anatomy of the modified anatomical structures in MSH; as such, it could be of help in a surgical approach.

## 1. Introduction

The maxillary sinus (MS, Highmore’s antrum) lies within the maxillary bone. It drains in the ethmoidal infundibulum of the lateral nasal wall which, in turn, is a narrow passage on the outer side of the uncinate process.

Bolger et al. (1990) defined the maxillary sinus hypoplasia (MSH) as an occasional “anomaly of the paranasal sinuses” [1], even when smaller the maxillary sinus retains a pyramidal configuration, although truncated [2]. Syndromal etiologies of MSH can be broadly divided into lack of development from failure of midfacial skeleton growth and obliteration of the sinus cavities from osteosclerosis [2]. Maxillary sinus hypoplasia may be associated with sinusitis due to the obstruction of the osteomeatal complex [3]. Unilateral MSH is more common than the bilateral condition in most studies [4].

Bassiouny et al. (1982) classified MSH as grade I—mild MSH with limited infero-lateral expansion—and grade II, which adds a curved orbital floor and lateral displacement of the lateral nasal wall encroaching on the antral lumen [5]. The compensatory enlargement of the nasal cavities in cases with MSH has also been observed by others [6].

Bolger proposed a three-type classification system of MSH [1]. In type I MSH, the uncinate process and ethmoidal infundibulum are normal and well defined, there are varying degrees of mucosal thickening within the hypoplastic sinus, and there is a mild hypoplasia. In type II, the uncinate process is hypoplastic or absent, the ethmoidal infundibulum is ill defined or absent, the hypoplastic sinus is completely opacified, and there is significant MSH. In type III, the uncinate process is absent and the maxillary sinus appears just as a shallow cleft in the lateral nasal wall. Sirikçi et al. (2000) added the orbital enlargement to the changes in types II and III of MSH defined by Bolger [7]. Selçuk et al. (2008) added several other possible modifications in MSH: bony thickening of sinus wall, hypoplasia or aplasia of other sinuses, canine fossa elevation, anterior ethmoidal cell variation, and presence of ethmomaxillary sinus [8,9]. Cases of MSH have also been detected with superior orbital fissure asymmetry [5] or enlargement [9], medial localization of the orbit [10], depression of the orbital roof [11], and lateral displacement of the infraorbital canal [9]. Posterior ethmoid air cells expanded within the MS are regarded as ethmomaxillary sinuses [12]. If such an ethmomaxillary sinus is associated with a MSH, the orbit is normal but the superior nasal meatus is enlarged [13].

We noticed that previous studies dealing with MSH did not detail the consequent anatomical changes of the maxilla and adjacent fossae; thus, we decided to do so in a case with bilateral MSH.

## 2. Materials and Methods

The archived Cone Beam Computed Tomography (CBCT) files of a 58-year-old female case were documented. An iCat CBCT machine (Imaging Sciences International, Hatfield, PA, USA) was used, with a resolution of 0.250, FOV of 130, and image matrix size of 640 × 640. The CBCT files were analyzed with Planmeca Romexis Viewer 3.5.0.R software, as in other previous studies [14,15,16]. Coronal, axial, and reconstructive panoramic sections and images were used. The files were additionally documented with Horos for iOS software (Horos Project), which was used for three-dimensional volume renderings. The patient gave written informed consent for use of medical data if anonymized. The study was conducted according to the guidelines of the Declaration of Helsinki, and was approved by the Ethics Committee of the second affiliation of author #4 (protocol code 456/04.05.2021).

## 3. Results

**Figure 1:**
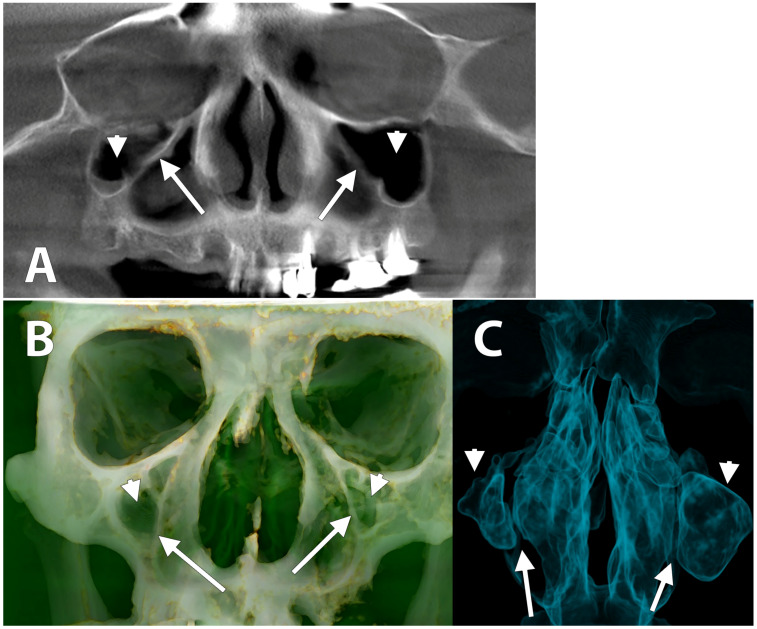
Bilateral maxillary sinus hypoplasia, anterior views. (**A**) Digital orthopantomogram; (**B**) three-dimensional volume rendering (Bone+Skin Filter); (**C**) three-dimensional volume rendering (Airways Filter). Hypoplastic maxillary sinuses (arrowheads) and the lateral nasal walls (arrows) are indicated.

**Figure 2:**
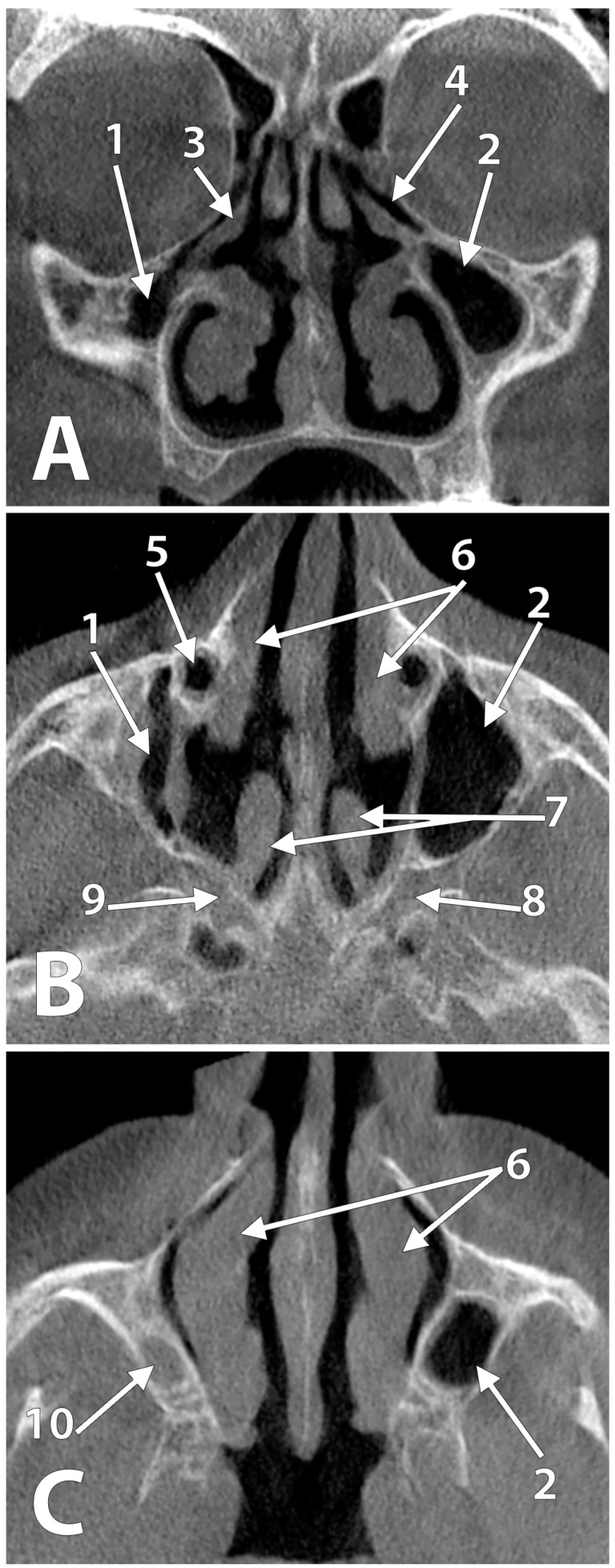
Planar CBCT slices through the hypoplastic maxillary sinuses. (**A**) Coronal slice through the second upper premolar regions; (**B**) axial slice through the roofs of the maxillary sinuses; (**C**) axial slice through the floor of the right maxillary sinus. (1) right maxillary sinus; (2) left maxillary sinus; (3) right uncinate process; (4) left ethmoidal infundibulum; (5) right aerated nasolacrimal duct; (6) inferior nasal turbinates; (7) middle nasal turbinates; (8) left pterygopalatine fossa; (9) right pterygopalatine fossa; (10) floor of the right maxillary sinus.

In the case we document here, an asymmetrical bilateral MSH was found (Figure 1). The maximum height of the right MS was 13.61 mm. The left MS maximum height was 27.96 mm. The maximum medio-lateral widths of the right and left MS were 8.27 mm and 17.26 mm, respectively. In both MSs, a mild thickening of the antral floor mucosa was found. A septum of the antral floor was found above the first left upper molar. Orbital enlargement was not detected on any side. However, the orbital floors were bilaterally curved (Figure 2A). There were no evident anatomical changes of the ethmoidal cells or frontal and sphenoidal sinuses. The upper part of the nasal septum had a minor right deviation of 1.25 mm.

The uncinate process and the ethmoidal infundibulum were anatomically normal. However, they were laterally displaced beneath the orbit floor. On the right side, the uncinate process was located inferior to the medial half of the orbit floor. Thus, the respective ethmoidal infundibulum was limited superiorly by the orbit floor and inferiorly by the uncinate process. On the left side, with a larger MS, the uncinate process was inferior just to the medial ¼ of the orbit floor. Therefore, the left ethmoidal infundibulum was inferior to the orbit floor and the respective ethmoidal bulla. As both uncinate processes were bent medially, they could have been regarded as accessory middle nasal turbinates.

On each side, the lateral nasal wall protruded within the respective maxillary bone to reach above the vestibular cortical plate of the alveolar process (Figure 2A). This lateral expansion of the lateral nasal walls was limited anteriorly. Therefore, the alveolar bone was beneath that nasal fossa in the premolar and first molar regions. Only in the second and third molar regions the alveolar base was beneath the hypoplastic antrum. On each side, the canine fossa was deepened above the premolar regions. The inferior turbinates’ free borders were at the level of the floors of the respective MS. On each side, the nasolacrimal ducts were aerated (Figure 2B) from the inferior nasal meatus. On axial slices (Figure 2C), the inferior turbinates appeared laterally curved. On each side, the perpendicular plate of the palatine bone was building a *bona fide* postero-lateral nasal wall separating that nasal fossa from the pterygopalatine fossa (Figure 2B) and attaching the twisted tail of the middle nasal turbinate. Therefore, the sphenopalatine foramen reached the same sagittal plane as the vidian canal.

## 4. Discussion

In this report, we present an asymmetrical bilateral MSH. According to Bolger’s classification system, it is a bilateral type I MSH, with morphologically unaltered uncinate process and ethmoidal infundibulum, as documented in CBCT. Various studies have used Bolger’s classification of MSH [2,3,4,8,10,17,18,19,20,21,22,23,24,25,26,27]. Numerous studies have documented MSH mostly, or exclusively, by coronal CT slices [2,6,8,17,18,19,22,23,26,27]. We evaluated the MSH in all three anatomical planes, as well as on three-dimensional renderings. Other CBCT studies of the maxillary sinus either only listed the MSH among other anatomic variations of the sinus [28] or did not mention MSH [29].

Here, the anatomical variables modified by MSH are documented. These were mostly overlooked in previous reports.

The classification systems should be further detailed. This is because in type I MSH, the uncinate process and ethmoidal infundibulum could either be normally placed in the lateral nasal wall or they could be displaced in variable degrees beneath the orbit floor, as was observed here.

We noticed that the lateral expansion of the lateral nasal wall in MSH was limited to the anterior part of that nasal wall, and was thus not complete sagitally. The laterally expanded nasal fossa reached between the canine fossa and the pterygopalatine fossa in this bilateral MSH.

The uncinate process is a key landmark in endoscopic sinus surgery [23]. The uncinate process could be absent in MSH; thus, the medial orbital wall could be penetrated during endoscopic procedures [26]. An uncinate process located beneath the orbit floor, such as in this case, leads to an increased risk of penetrating the orbit floor during endoscopic procedures.

Maxillary sinus hypoplasia can simulate sinusitis and other conditions [30]. Due to an inaccurate diagnosis, unnecessary medications or surgical procedures could occur [30].

In cases of MSH with lateral expansion of the nasal fossa, anterior to the pterygopalatine fossa, this could later be easily approached via the middle nasal meatus through a transnasal (rather than transmaxillary) endoscopic corridor.

In the case of MSH, endodontic and surgical treatment is less likely to cause complications due to a larger distance between the teeth apices and the antral floor [31].

On other hand, a laterally expanded nasal fossa reaches above the maxillary alveolar bone, thus above the teeth that are normally regarded as located just beneath the antral floor. The nasal cavity pneumatization in the posterior direction was indicated as an inferior meatus pneumatization [32]. It can extend up to the second molar area and limits the available height of the residual ridge [32]. Therefore, inserting endosseous dental implants without a careful CBCT check could result in oronasal iatrogenic penetration. In this regard, a case with MSH was previously reported in which the nasal floor was resorbed and the corresponding nasal mucosa was thickened by a periapical periodontitis of the first maxillary molar [33].

When MSH is detected on CT or CBCT scans, other paranasal pneumatizations should also be checked. A case was previously reported in which bilateral maxillary and ethmoidal sinus aplasia were combined with aplasia of the frontal and sphenoidal sinuses [25]. A post-Caldwell-Luc MSH should be distinguished from a congenital MSH [34]. Congenital MSH could occur with other paranasal sinuses anomalies [35]. In the case presented here, neither previous Caldwell-Luc Surgery nor other paranasal sinuses anatomic changes were found.

The silent sinus syndrome is underestimated in imagistic reports [36]. The long-term hypoventilation of MS results in negative pressure within the sinus. It usually manifests clinically as hypoglobus and enophthalmos, associated with ipsilateral MS atelectasis [37,38]. While the superior, lateral, and nasal walls of atelectatic MS protrude within the sinus in the silent sinus syndrome, the alveolar bone height is not augmented consistently, such as in MSH.

## 5. Conclusions

We conclude that CBCT is a better tool than CT for evaluating the detailed anatomy of the modified anatomical structures in MSH; as such, it could be of help in a surgical approach.

## Data Availability

No new data were created or analyzed in this study. Data sharing is not applicable to this article.
